# Bonding Mechanical Properties between SMA Fiber and ECC Matrix under Direct Pullout Loads

**DOI:** 10.3390/ma16072672

**Published:** 2023-03-28

**Authors:** Zhao Yang, Xiaojun Gong, Qing Wu, Lin Fan

**Affiliations:** 1 School of Urban Construction, Wuhan University of Science and Technology, Wuhan 430065, China; 2 Hubei Provincial Engineering Research Center of Urban Regeneration, Wuhan University of Science and Technology, Wuhan 430065, China

**Keywords:** shape memory alloy fiber, superelasticity, engineered cementitious composites, bonding performance

## Abstract

SMAF-ECC material composed of shape memory alloy fiber (SMAF) and engineered cementitious composite (ECC) has good bending and tensile properties, as well as good crack self-healing ability, energy consumption, and self-centering ability. The bond behavior between fiber and matrix is crucial to the effective utilization of the superelasticity of SMAF. The experimental study considered three variables: SMA fiber diameter, fiber end shape, and bond length. The pullout stress–strain curve of SMAF was obtained, and the maximum pullout stress, maximum bond stress, and fiber utilization rate were analyzed. Compared with the straight end and the hook end, the maximum pullout stress of the specimen using the knotted end SMAF is above 900 MPa, the fiber undergoes martensitic transformation, and the fiber utilization rate is above 80%, indicating that the setting of the knotted end can give full play to the superelasticity of the SMAF. Within the effective bond length range, increasing the bond length can increase the maximum anchorage force of the knotted end SMAF. Increasing the fiber diameter can increase the maximum pullout stress and maximum anchoring force of the knotted end SMAF but reduce the utilization rate of SMA fiber. This study provides a reliable theoretical basis for the bonding properties between SMAF and ECC.

## 1. Introduction

Traditional concrete materials have poor tensile properties, poor impact toughness, obvious strain softening characteristics, and large crack width, making the internal steel susceptible to corrosion [1,2]. In order to meet the requirements of modern construction and overcome the defects of traditional concrete, various types of high-performance fibers were combined with concrete materials to develop high-performance fiber-reinforced concrete materials. In the early 1990s, Engineered Cementitious Composites (ECC) were successfully developed at the University of Michigan [3]. ECC is a kind of cementitious composite material reinforced by randomly distributed short fibers which has ultra-high toughness, high tensile strength, and high fracture resistance [4,5,6,7,8,9]. Different from ordinary concrete and fiber-reinforced concrete materials, ECC will undergo strain hardening after it cracks. The tensile strain capacity of ECC can be 300 to 500 times that of ordinary concrete, and it will produce a large number of small and evenly distributed cracks before it breaks [10]. In addition, ECC has high shear ductility, energy dissipation capacity, and damage tolerance, so it is widely used to improve the seismic performance of structures. Maalej et al. [11] and Yuan et al. [12] found that ECC beams had high bearing capacity, high ductility, high energy consumption, and plump hysteresis loop. Shan et al. [13] found that the ultimate bearing capacity, ductility, and crack resistance of ECC columns was significantly improved compared with concrete columns. Yuan et al. [14] verified that replacing concrete with ECC in the beam–column joint region could significantly improve the bearing capacity, ductility, energy dissipation capacity, and shear strength of the beam–column joints. However, ECC will produce significant residual deformation and a large number of crack damage, which is still not conducive to the structural repairing or strengthening [15].

**Figure 1:**
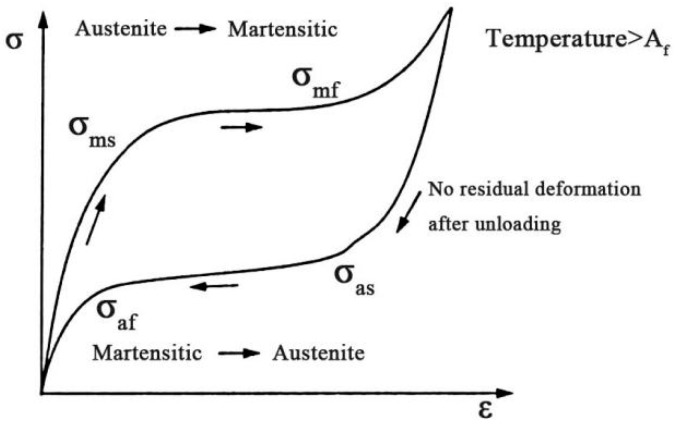
Schematic diagram of SMA superelasticity.

Shape memory alloy (SMA) is a kind of functional material with special shape memory effect and superelasticity which has been used in aerospace, automobile, robot, biomedicine, civil engineering, and other fields [16,17,18,19,20]. Superelasticity is a characteristic of austenitic SMA material produced by stress excitation at normal temperature. As shown in Figure 1, σ_ms_ and σ_mf_ are the starting and ending stress of martensitic transformation separately, σ_as_ and σ_af_ are the beginning and ending stress of austenite transformation, respectively, and A_f_ is the temperature at the end of austenite transformation. SMA material can produce an obvious energy dissipation loop during the loading and unloading process, and the strain can be significantly recovered after unloading [21,22]. Therefore, superelastic SMA can be used in seismic structures to improve the energy dissipation capacity and provide self-centering capacity for structures. Pei et al. [23] found that SMA bars were able to improve the bearing capacity, ductility, and self-centering capability of concrete beam–column joints. Cortés-Puentes et al. [24] proved that the shear walls strengthened by SMA rods had a self-centering ability and were able to self-repair the damaged areas. However, in these studies, SMAs are mainly fabricated into rods, bars, or strands. These continuous SMA products are expensive, difficult to process, need special connectors to connect with steel bars, and are prone to weak cross-sections, which seriously affect the application and promotion of SMA materials.

Therefore, short and randomly distributed SMA fibers were used instead of the above continuous products in mortar and concrete structures. However, the bonding strength between the SMA fibers and the mortar or concrete matrix is very poor due to the smooth surface of SMA fibers [25]. Therefore, different end shapes for SMA fibers were used to improve the bonding performance. Dehghani et al. [26] found that SMA fibers with 45° hook end showed a high bond strength with the concrete matrix, while Choi et al. [27,28] found that spearhead end provided a much greater pullout resistance for SMA fibers and showed a good flag-shaped energy dissipation behavior. They [29,30] also indicated that the larger diameter of SMA fibers, the better self-centering capacity of the cementitious composites. Ho et al. [31] found that the crimped fiber with a larger diameter provided a great peak pullout stress. However, due to the brittleness of concrete or mortar, they are easily damaged in tension, so the performance of SMA cannot be fully utilized. 

Compared with concrete or mortar, ECC has much larger tensile deformation capacity, so it is more compatible with SMA fibers. In addition, the crack distribution of ECC is wide and the crack width is small, which can enable more SMA fibers to participate in the work and reduce the difficulty of SMA fibers to close the crack [32]. On the other hand, SMA fibers can provide crack closure and self-centering capability for ECC, which can effectively solve the problems of residual deformation and residual cracks of ECC materials. Ali et al. [33] found that compared with ECC made with only 2% PVA by volume fraction, the ECC beams strengthened with SMA fibers obtained higher flexural strength, increased by 39%, and the self-centering rate reached 36%. Dehghani et al. [34,35] found that SMA fibers could improve the flexural toughness and tensile properties of ECC materials. Chen et al. [36] also proved the improvement in strength and ductility and the self-centering ability of SMA-ECC specimens. The author’s previous research [32] also confirmed the effectiveness of SMA fibers in improving the energy dissipation and self-centering ability of ECC beams.

Although the incorporation of SMA fibers into ECCs has yielded exciting results, it is evident that the prerequisite for the full utilization of SMA fiber’s superelasticity is its sufficient bond strength with ECC. However, just like concrete and mortar matrix materials, the bonding strength of ECC matrix and SMA fiber is very poor because of the smooth surface of SMA fibers, so it is necessary to take some measures to improve the bonding or anchoring strength of SMA fiber and ECC matrix. In the previous study, the author studied the deformation recovery and cyclic energy dissipation ability of SMA fibers and ECC matrix under cyclic pullout loads [37]. In order to more accurately analyze the bonding and anchoring capacity of SMA fiber and ECC matrix, the strength utilization ratio of SMA fiber before anchoring failure, the direct pull-out test of SMA fiber and ECC matrix was carried out in this study, and the influencing factors such as end shape, diameter, and bonding length of SMA fibers were compared as well. This study can provide a reliable basis for improving the bonding capability of SMA fiber and ECC matrix, conducting theoretical research on the mechanism of SMA fibers in ECC matrix.

## 2. Experiment Design

### 2.1. Materials

**Figure 2:**
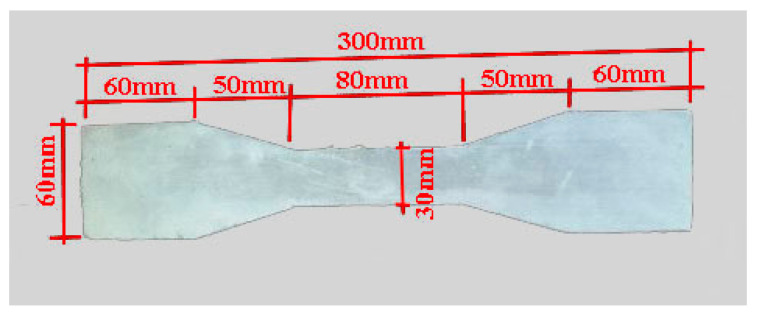
ECC dumbbell-shaped tensile specimen.

In this test, the main components of ECC include Type I ordinary Portland cement. The measured compressive strength after 28 days of curing was 42.5 MPa; high quality fly ash, fineness was 43 μm, density was 2.4 g/cm^3^, and water content was 0.5%; for white crystalline quartz sand, fineness was 100~200 mesh; for PVA fibers, length was 9 mm, diameter was 31 μm, tensile strength was 1500 MPa, elastic modulus was 42 GPa, and elongation was 6%; polycarboxylate is a high performance water-reducing agent. The experimental mix proportion of ECC is shown in Table 1. According to Chinese Standard JC/T 2461-2018: Standard test method for the mechanical properties of ductile fiber-reinforced cementitious composites [38], the dumbbell-shaped specimens were made to test the tensile properties of ECC material, and the specified size of tensile specimen is shown in Figure 2. The thickness of ECC specimen is 13 mm. 

**Figure 3:**
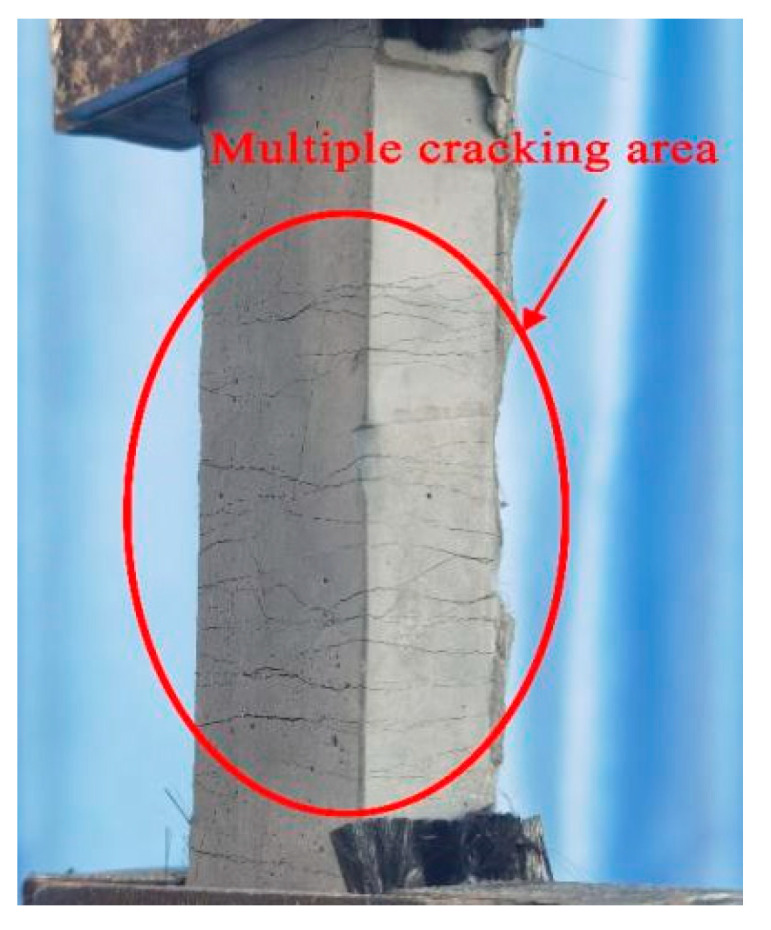
The cracking diagram of ECC.

**Figure 4:**
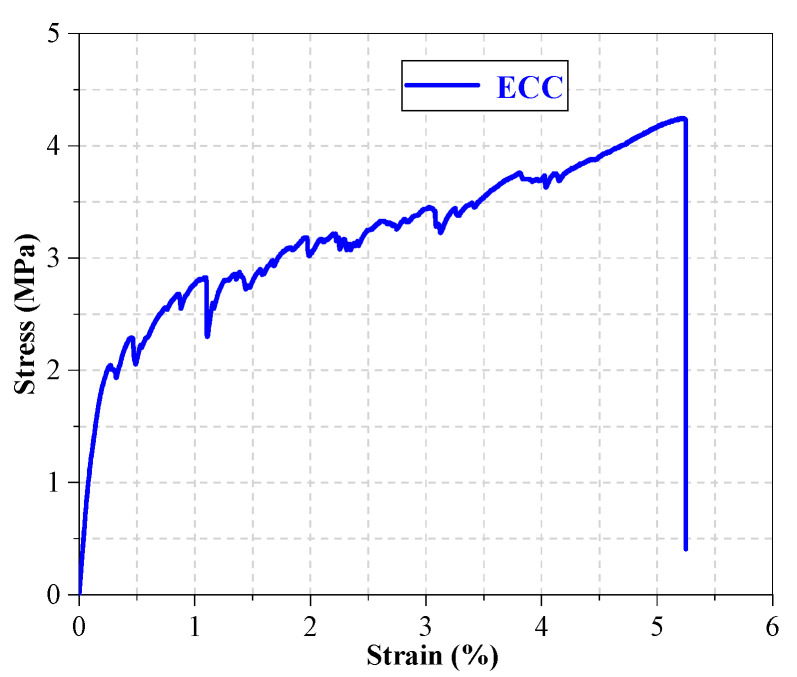
The tensile stress–strain curve of ECC.

**Figure 5:**
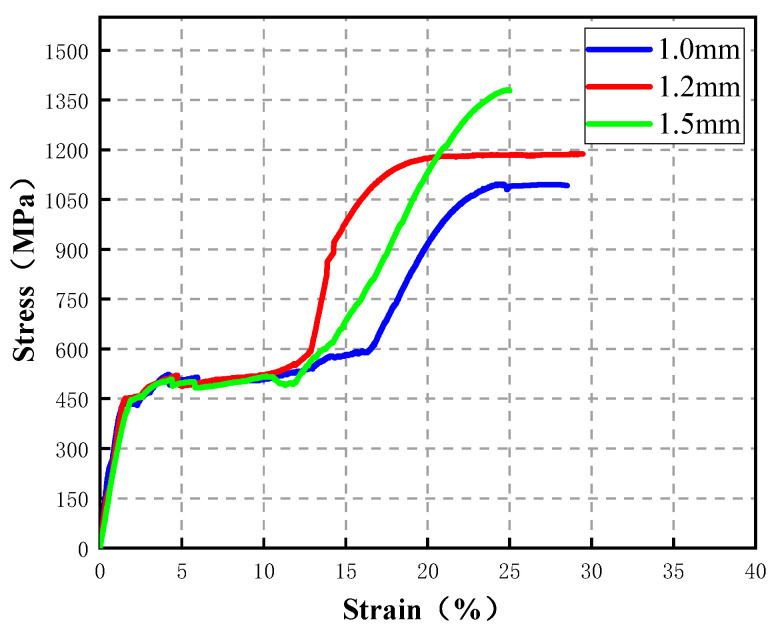
Direct tensile test stress–strain curves of SMA.

In the test, obvious multiple cracks were observed, as shown in Figure 3. From Figure 4, ECC showed obvious strain hardening characteristics after cracking, the ultimate strain (the corresponding strain when the stress drops to 85% of the peak stress) reached 3.12%, and the peak tensile stress reached 4.25 MPa. In addition, the curve fluctuated obviously, and each fluctuation indicates a new crack appeared in the specimen [39]. Nickel–titanium superelastic SMA fibers were used in this test, with diameters of 1.0 mm, 1.2 mm, and 1.5 mm, respectively. Direct tensile tests were carried out by a universal testing machine to obtain the main mechanical performance of these SMA fibers. The main mechanical performance of the SMA fibers is shown in Table 2 and the stress–strain curves are shown in Figure 5.

### 2.2. Specimen Design and Fabrication

**Figure 6:**
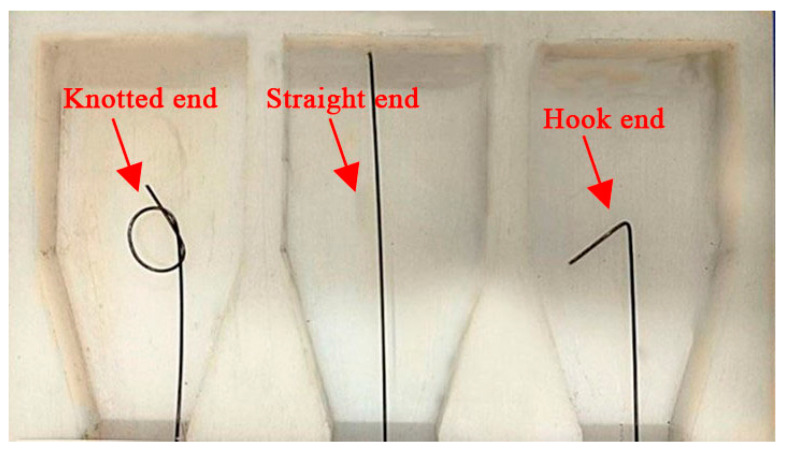
SMA fiber end shapes.

**Figure 7:**
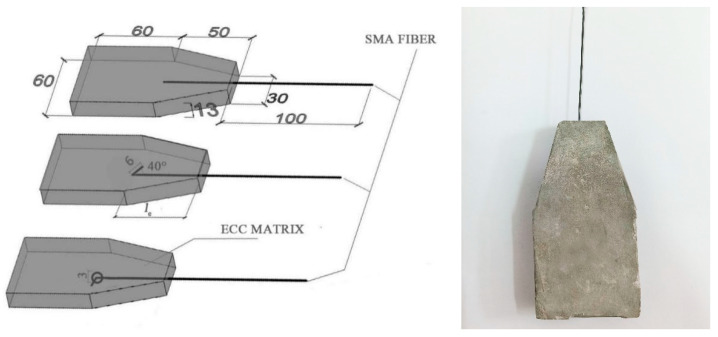
Specific size of specimen. l_e_ is the bonding length, from top to bottom are straight specimens, hook specimens, and knotted specimens.

In order to study the bonding properties of SMA fiber and ECC matrix, direct pullout tests of SMA-ECC specimens were carried out, and the SMA fiber diameter, end shape, and bond length were taken as the main influencing factors. The diameters of the SMA fiber are 1 mm, 1.2 mm, and 1.5 mm, respectively. The end shapes are set to be straight, hook, and knotted, as shown in Figure 6. The bonding length was set to be 30 mm, 40 mm, and 50 mm, not including the length of hook or knot. The grouping table of the test specimens is shown in Table 3. The specific dimensions of the specimens are shown in Figure 7. The ECC mix ratio used in the tests is the same as Table 1. After being fabricated, the specimens were placed into a standard curing box, demolded after 24 h, and put into the water tank to continue standard curing for 28 days.

### 2.3. Test Device and Test Method

**Figure 8:**
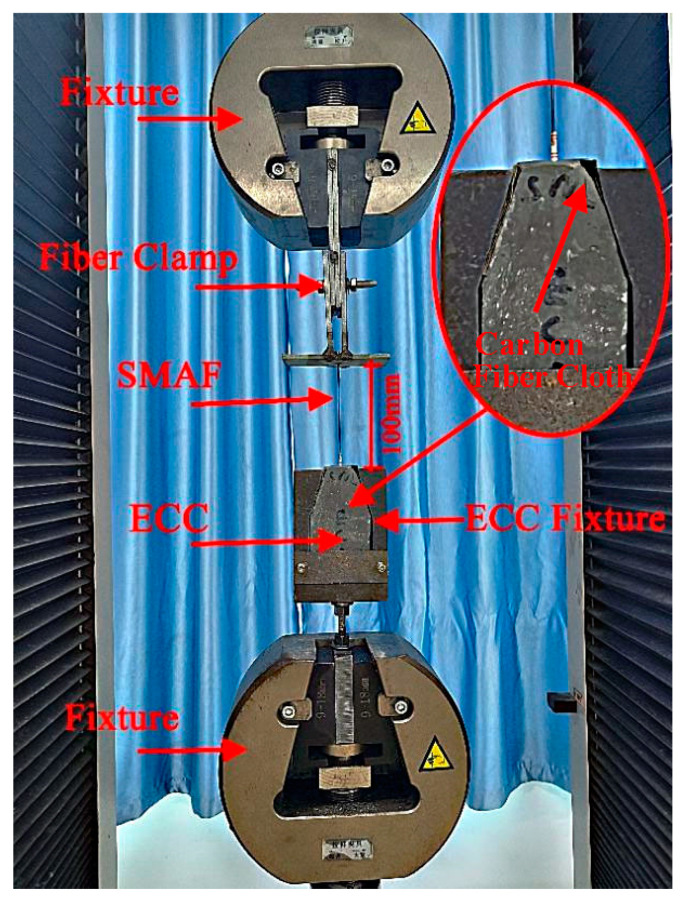
Test devices.

A universal testing machine was used for the direct pullout test, and the loading speed was controlled by the pulling displacement as 2 mm/min. The loading was stopped when the fiber was broken or pulled out. In order to reduce the slip between the ECC matrix and the fixture of the testing machine, a specific ECC fixture was used to fix the matrix, and the carbon fiber cloth was used to wrap the specimen to reduce the stress concentration of the fixture. A specific fiber clamp was used to fix the free end of the SMA fiber. The length of the free tensile section was 100 mm. The built-in sensor of the testing machine was used to record the load and the pulling displacement during the test. The test data were synchronously collected by the computer. The test devices are shown in Figure 8. The pull-out test analyzed the bonding behavior of SMA fiber and ECC matrix, the bond failure process, and the stress level of SMA fibers. Therefore, this study analyzed the principal indicators such as load, displacement, and SMA fiber stress, and expressed them by the load–displacement–stress combination curve. On this basis, the bond-bearing capacity and SMA fiber strength utilization ratio was compared.

## 3. Results of Pullout Tests

### 3.1. Pullout Mechanical Properties

**Figure 9:**
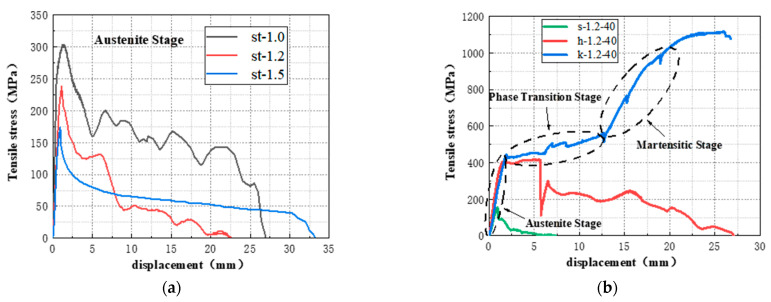

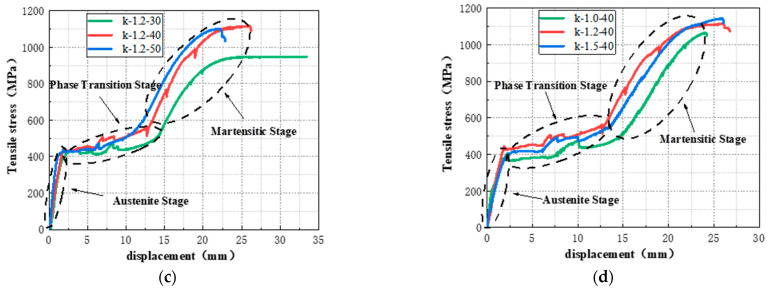
The pullout load-displacement-stress curves of test specimens. (**a**) The first group; (**b**) The second group; (**c**) The third group; (**d**) The fourth group.

**Figure 10:**
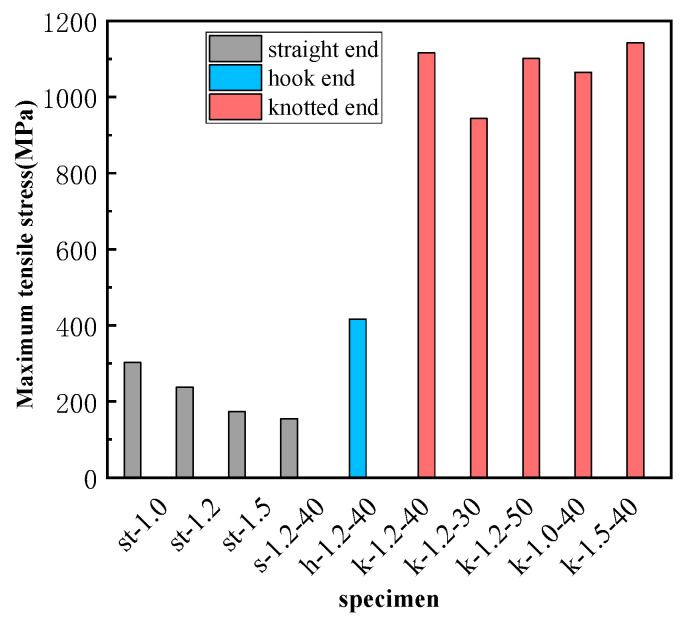
Comparison of the maximum pullout stress of each specimen.

The pullout load–displacement–stress curves of test specimens are shown in Figure 9, and the main pullout mechanical parameters are shown in Table 4. In Table 4, *σ_f_* is calculated according to Equation (1).

(1) $$\sigma_{f}=\frac{F}{A_{f}}=\frac{F}{\pi \cdot \frac{d_{f}^{2}}{4}}$$

where *σ_f_* is the pullout stress of the SMA fiber; *F* is the pullout load; *A_f_* is the section area of the SMA fiber; *d_f_* is the diameter of the SMA fiber. The comparison of the maximum pullout stress of the SMA fiber in each specimen is shown in Figure 10.

#### 3.1.1. The First Group

**Figure 11:**
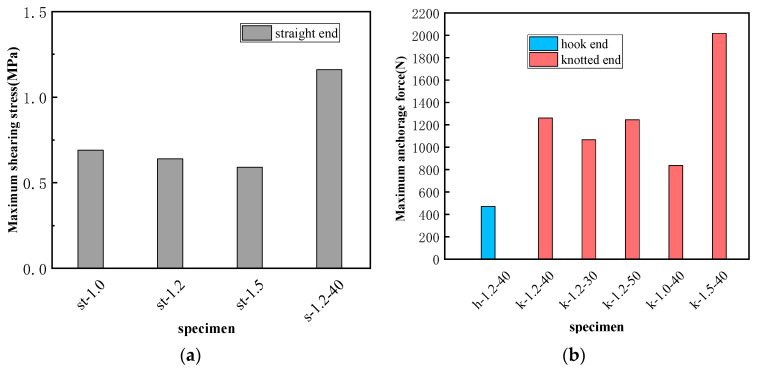
The comparison figure of the calculation parameters of test specimens. (**a**) Bond strength of SMA fibers with straight end; (**b**) Maximum anchorage force of SMA fibers with hook and knotted end.

The specimens in this group include st-1.0, st-1.2, and st-1.5, and SMA fibers in these specimens have the same straight end and bond length, but different diameters. It can be seen from Figure 9a that when the loading displacement is small, the curves are approximately linear. After reaching the peak point, the pullout stress of the fiber decreases rapidly, and the displacement increases significantly. From Table 4, the maximum pullout stress of the SMA fibers was only 302 MPa (st-1.0), 238 MPa (st-1.2), and 173 MPa (st-1.5), respectively, which was far less than the corresponding phase transformation platform stress shown in Table 2. Therefore, the martensitic transformation of SMA fibers cannot be induced. This is due to the smooth surface of SMA fiber that led to very small chemical cemented force and friction force, and the straight end provided no anchoring force. Larger diameters can result in reduced stress, as shown in Figure 11. The reason is that the Poisson effect in the pulling process decreases the SMA fiber diameter, thus making the bonding surface more prone to failure and thereby reducing the pullout stress of the fiber [40].

#### 3.1.2. The Second Group

The specimens in this group include s-1.2-40, h-1.2-40, and k-1.2-40, and SMA fibers in these specimens have the same diameter and bond length but different end shapes. From Figure 9b and Table 4, the SMA fiber with a straight end has a very small maximum pullout stress of 155 MPa, and there is no phase transformation stage. As for the hook end fiber, a short phase transformation process can be observed, but there is no martensitic hardening stage. The maximum pullout stress is 416 MPa and can start the phase transformation, so the anchoring performance of the hook end is better than the straight end [41]. However, due to the stress concentration, the bending hook is fractured and leads to an anchorage failure. Therefore, the hook fiber could not withstand higher stress and could not fully produce superelasticity. For the knotted end fiber, the curve has an obvious phase transformation stage and hardening stage. The maximum pullout stress of this SMA fiber reaches 1116 MPa, which is 3.38 and 2.68 times of the stress of straight end fiber and the hook end fiber separately. It proves that the knotted end can provide enough anchoring force for SMA fiber, resulting in superelasticity and effectively reducing slip.

#### 3.1.3. The Third Group

The specimens in this group include k-1.2-30, k-1.2-40, and k-1.2-50, and SMA fibers in these specimens have the same diameter and end shape but a different bond length. As shown in Figure 9c, SMA fibers in these specimens can all experience the phase transformation and martensitic hardening stage before being fractured. From Table 4 and Figure 10, the maximum pullout stresses are 943 MPa (k-1.2-30), 1116 MPa (k-1.2-40), and 1101 MPa (k-1.2-50), which shows that longer bond length results in higher pullout stress. However, as to the maximum displacement, bonding length has an opposite effect. It is because longer bond length can provide greater frictional resistance between SMA fiber and ECC matrix. Therefore, the pullout stress can be increased, and the slip can be decreased. The maximum pullout stresses of the SMA fibers in k-1.2-40 and k-1.2-50 are very close, which is because there is an effective bond length between SMA fiber and ECC matrix when the bond length is close to or exceeds the effective bond length, and the interfacial friction cannot continue to increase with the increase of bond length [42].

#### 3.1.4. The Fourth Group

The specimens in this group include k-1.0-40, k-1.2-40, and k-1.5-40, and SMA fibers in these specimens have the same bond length and end shape but different diameters. Figure 9d shows that all the SMA fibers can enter the martensitic hardening stage before fractured. From Table 4 and Figure 10, the maximum pullout stress is 1064 MPa (k-1.0-40), 1116 MPa (k-1.2-40), and 1142 MPa (k-1.5-40), showing that larger fiber diameter leads to higher maximum pullout stress, which is different with the fibers in the first group. This is because knotted ends can provide significant anchorage force, especially at the ultimate state, and the bond-bearing capacity of the knotted end fiber mainly comes from the anchorage force. As the fiber diameter increases, the anchoring force increases, resulting in higher pullout stress.

### 3.2. Bond-Bearing Capacity of SMA Fibers

#### 3.2.1. Calculation Model of Bond-Bearing Capacity

**Figure 12:**
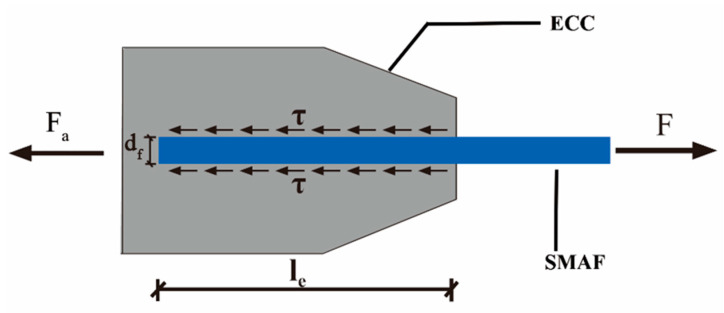
The force balance diagram of the SMA fiber during the pullout process.

Figure 12 shows the force balance diagram of the SMA fiber during the pullout process. Therefore, the relationship between pullout load F and average interfacial bonding stress τ and anchoring force *F_a_* can be established as in Equation (2).

(2) $$F=\tau \pi d_{f}l_{e}+F_{a}$$

where *d_f_* is the same as Equation (1) and *l_e_* is the bond length.

As for SMA fibers with straight ends, the bond strength mainly comes from interfacial bonding stress and there is no anchoring force. Thus, the average bonding stress can be calculated through Equation (3) [43].

(3) $$F=\tau \pi d_{f}l_{e}$$

As for SMA fibers with hook end and knotted end, the bond strength mainly comes from the anchoring force *F_a_*. With the increase of the pullout load, the interface between SMA fiber and ECC matrix begins to debond, and the debonding area develops from the loading end to the fiber end. When the interface along the bonding length is completely debonded, the pullout load is mainly resisted by the anchoring force of the fiber end. Therefore, in the ultimate state of bearing capacity, the maximum pullout load can be expressed as in Equation (4).

(4) $$F=F_{a}$$

#### 3.2.2. Calculation Result and Comparison Analysis

Through Equation (3) and the maximum pullout load obtained in the test, the average bond strength τ_max_ of SMA fibers with straight end can be calculated. Similarly, by Equation (4) and the tested data, the maximum anchoring force *F_a_* of SMA fibers with hook and knotted end can be calculated as well. The calculated parameters of bond-bearing capacity are shown in Table 5, and the comparison of the calculation parameters is shown in Figure 11. As for SMA fibers with straight end, when the bond length is all 110 mm (st-1.0, st-1.2, st-1.5), the larger the diameter, the lower the bond strength. The reason is same as the effect on pullout stress. For specimen s-1.2-40, the bond length is much smaller than the above three specimens, but its bond strength is much higher. This phenomenon proves that there must be an effective bond length as we discussed in Section 3.1.3, which is smaller than 110 mm. Therefore, when we calculated the average bond strength according to the bond length of 110 mm, the calculated value will be too small. As for SMA fibers with hook end, the maximum anchorage force is 471 N, which is far smaller than the fibers with knotted end. Just like the analysis in Section 3.1.2, the straightened or broken of the hook end can not provide enough anchorage force for the SMA fiber to undergo full phase transformation [44,45]. As for SMA fibers with a knotted end, the anchoring force is much higher than the hook end fibers and can ensure that the pullout stress of SMA continues to grow until it enters the martensitic hardening stage, thereby giving full play to the superelastic properties of the material. From Figure 11b, the anchoring force is very close when the bond length exceeds 40 mm, while the fiber diameter has a significant effect on the anchoring force. The maximum anchoring force 2017 N comes from the SMA fiber with 1.5 diameter, even though its bond length is only 40 mm. These influence laws are consistent with those of pullout stress, as discussed in Section 3.1.3 and Section 3.1.4.

### 3.3. Utilization Rate of SMA Strength

**Figure 13:**
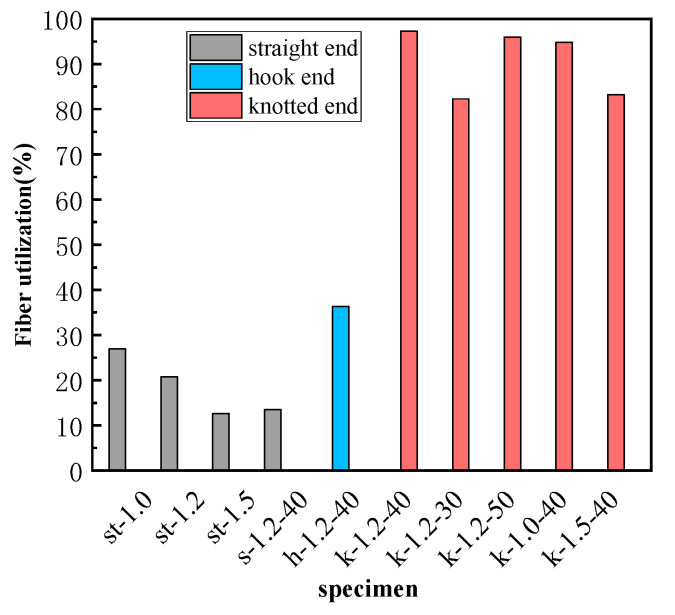
The comparison figure of the SMA strength utilization rate of test specimens.

In order to qualify the utilization of SMA strength, the coefficient *u_f_* was used, and the specific expression is shown in Equation (5).

(5) $$u_{f}=\frac{\sigma_{f,max}}{f_{y}}\cdot 100\%$$

where *f_y_* is the tensile strength of the SMA fiber. When *u_f_* is greater than 100%, it represents that the fiber has been fractured [43]. The SMA strength utilization rate of each specimen is shown in Table 6, and the comparison figure is shown in Figure 13.

For straight end SMA fibers, the utilization rates are very low, and are less than 30%. The influence of diameter and bond length on the strength utilization rate of SMA fiber is consistent with the influence of pullout stress and bond-bearing capacity. The strength utilization rate of the hook end SMA fiber is only 36.3%, further explaining that the hook end cannot effectively utilize the superelasticity of the SMA material. For knotted end SMA fibers the strength utilization ratio can be more than 80%, and the highest fiber utilization ratio comes from specimen k-1.2-40, which reaches 97.2%. Comparing specimens k-1.2-30, k-1.2-40, and k-1.2-50, which are different in bond lengths, their SMA fiber utilization rates are 82.2% (k-1.2-30), 97.2% (k-1.2-40), and 95.9% (k-1.2-50), respectively. It shows that the effect of bond length on strength utilization keeps accordance with that of pullout stress and bond capacity. However, the effect of fiber diameter is different. The strength utilization rates of specimen k-1.0-40, k-1.2-40, and k-1.5-40 are 94.8%, 97.2%, and 83.2%, respectively. It shows that as the diameter of SMA fiber increases, the utilization rate of fiber decreases. This is because larger diameter SMA fibers have higher tensile strength in the test, while the maximum pullout stress growth rate with the diameter increase is lower than the increase in material strength, resulting in the decrease of strength utilization. This result provides a reference for reasonable selection of fiber diameter. It is worth noting that although this study is backed up by relevant literature to support the conclusions, the limited number of samples in this experiment raises concerns about the validity of the conclusions. Therefore, in subsequent studies, further increasing the sample size is necessary to confirm the rationality of the conclusions.

## 4. Summary and Conclusions

In this paper, the bonding performance between SMA fiber and ECC was studied through the direct pullout tests. The fiber pullout mechanical properties, bond-bearing capacity and SMA strength utilization were analyzed, and the SMA fiber end shape, diameter, and bond length were compared as well. This study can provide a basis for the application of this new material in practical engineering. The main conclusions are as follows:

1. By setting the proper shaping of the end anchorage, the bonding performance between SMA fiber and ECC matrix can be effectively improved, which provides the basic conditions for making full use of the superelasticity of SMA material.
2. The use of a knotted end in SMA fibers provides sufficient anchoring force to ensure the full utilization of SMA superelasticity. The pullout stress of the knotted end SMA fiber can reach a maximum value of 1116 MPa, which is significantly greater than the martensitic transformation stress. Furthermore, the maximum anchoring force can reach 2017 N, and the fiber strength utilization rate exceeds 80%.
3. Straight end and hook end cannot provide sufficient bond strength or anchoring force. Due to the anchoring force provided by the hook end, the pullout stress can reach the martensitic transformation start stress. However, the stress concentration at the hook results in the anchorage failure, and thus the superelasticity of SMA can not be fully used.
4. For SMA fibers with knotted end, increasing the fiber diameter can significantly increase the anchoring force, thus obtained higher pullout stress, but the fiber strength utilization rate will decrease. In addition, properly increasing the bond length can also increase the anchorage force, but there is an effective bond length between SMA fiber and ECC matrix. When the bond length is close to or exceeds the effective bond length, the anchorage force cannot obviously increase. Fiber bond length is recommended to be controlled at around 40 mm.

## Figures and Tables

**Table 1:** Mixture weight proportion of the ECC specimens [37].

Raw Materials	Cement	Fly Ash	Silica Sand	Water	PS	PVA * (%)
Mix proportion	1.0	2.4	0.36	0.26	0.0082	2.0

* Percentage of fiber content by volume.

**Table 2:** The main mechanical performance of the SMA fibers with different diameters.

Diameter/mm	Start Point of Stress Platform		End Point of Stress Platform		Tensile Strength ^a^/MPa	Ultimate Strain ^b^/%
	Strain/%	Stress/MPa	Strain/%	Stress/MPa		
1.0	1.55	450	17.22	591	1123	28.6
1.2	1.65	468	13.7	581	1147	29.1
1.5	2.08	459	12.5	520	1372	25.4

^a^ The ultimate tensile strength is taken as the peak stress in the direct tensile stress–strain curve of SMA. ^b^ The ultimate tensile strain is the strain corresponding to the fracture point in the direct tensile stress–strain curve of SMA.

**Table 3:** Test specimen grouping table.

Group	Specimen	Diameter (mm)	End Shape	Bonding Length (mm)
1	st-1.0	1.0	Straight	110 (through)
	st-1.2	1.2		
	st-1.5	1.5		
2	s-1.2-40	1.2	Straight	40
	h-1.2-40		Hook	
	k-1.2-40		Knotted	
3	k-1.2-30	1.2	Knotted	30
	k-1.2-40			40
	k-1.2-50			50
4	k-1.0-40	1.0	Knotted	40
	k-1.2-40	1.2		
	k-1.5-40	1.5		

**Table 4:** The main pullout mechanical parameters of test specimens ^a^.

Group	Specimen	F_max_/N	σ_f,max_/MPa	D_max_/mm
1	st-1.0	237	302	26.3
	st-1.2	269	238	23.3
	st-1.5	305	173	31.5
2	s-1.2-40	175	155	7.41
	h-1.2-40	471	416	27.2
	k-1.2-40	1261	1116	27.1
3	k-1.2-30	1067	943	33.9
	k-1.2-40	1261	1116	27.1
	k-1.2-50	1244	1101	23.6
4	k-1.0-40	835	1064	25.0
	k-1.2-40	1261	1116	27.1
	k-1.5-40	2017	1142	25.8

^a^ F_max_ is the maximum pullout load; σ_f,max_ is the maximum pullout stress of SMA fibers; D_max_ is the maximum displacement of SMA fibers.

**Table 5:** Calculated parameters of the bond-bearing capacity of test specimens.

Specimen	*d_f_*/mm	*l_e_*/mm	τ_max_/MPa	*F_a_* (N)
st-1.0	1.0	110	0.69	/
st-1.2	1.20	110	0.64	/
st-1.5	1.50	110	0.59	/
s-1.2-40	1.20	40.0	1.16	/
h-1.2-40	1.20	40.0	/	471
k-1.2-40	1.20	40.0	/	1261
k-1.2-30	1.20	30.0	/	1067
k-1.2-50	1.20	50.0	/	1244
k-1.0-40	1.00	40.0	/	835
k-1.5-40	1.50	40.0	/	2017

**Table 6:** The SMA strength utilization rate of test specimens.

Specimen	*f_y_* (MPa)	*u_f_* (%)
st-1.0	1123	26.9
st-1.2	1147	20.7
st-1.5	1372	12.6
s-1.2-40	1147	13.5
h-1.2-40	1147	36.3
k-1.2-40	1147	97.2
k-1.2-30	1147	82.2
k-1.2-50	1147	95.9
k-1.0-40	1123	94.8
k-1.5-40	1372	83.2

## Data Availability

Not applicable.
